# Omega-3 Fatty Acid Fortification of Flax Through Nutri-Priming

**DOI:** 10.3389/fnut.2021.715287

**Published:** 2021-08-20

**Authors:** Edward Marques, Heather Darby, Jana Kraft

**Affiliations:** ^1^The Department of Animal and Veterinary Sciences, The University of Vermont, Burlington, VT, United States; ^2^The Department of Plant and Soil Sciences, The University of Vermont, Burlington, VT, United States; ^3^The Department of Medicine, Division of Endocrinology, Metabolism and Diabetes, The University of Vermont, Colchester, VT, United States

**Keywords:** agronomic, functional food, biofortification, eicosapentaenoic acid, docosahexaenoic acid, alpha-linolenic acid

## Abstract

Omega-3 (n-3) fatty acids (FA) play an essential role in human physiology and health. As a result, a variety of n-3 FA-fortified functional foods have become commercially available for human consumption. These fortified functional foods are created through various processes; however, nutri-priming, a potentially promising fortification approach, has not been utilized to develop plant-based n-3 fortified foods. We sought to determine whether nutri-priming is a viable option to enrich seeds and sprouts with n-3 FA. Additionally, we assessed whether n-3 FA nutri-priming would inhibit germination of the primed seeds. To address these goals, we nutri-primed brown flax in three priming solutions, control [0% fish oil (FO)], 10% FO and a 20% FO solution, and determined the FA content and profile of seeds and sprouts and germination percentage of primed seeds. n-3 FA nutri-priming with FO altered the FA profile in seeds and sprouts, with increases in the absolute content of 20:5 n-3, 22:6 n-3, 22:5 n3, 18:4 n-3, and 20:4 n-6. However, n-3 FA nutri-priming did not increase the absolute content of 18:2 n-6, 18:3 n-3, total saturated FA, total monounsaturated FA, total polyunsaturated FA, total n-6 FA, or total n-3 FA. Our results also showed that n-3 nutri-priming decreased the germination percentage of primed seeds, with 10 and 20% FO priming solution reducing germination by 4.3 and 6.2%, respectively. Collectively, n-3 nutri-priming modified the n-3 FA profile in flax; however, the process does not increase the total n-3 FA content and inhibits germination of primed seeds. Further research utilizing different seed types, oil types, and oil concentrations needs to be conducted to fully determine if n-3 nutri-priming is a commercially viable approach for n-3 fortification of seeds and sprouts.

## Introduction

Omega-3 (n-3) fatty acids (FA) are polyunsaturated FA (PUFA) that play an important role in human physiology and health (1). n-3 FA are key components of the cell membrane's phospholipid bilayer, which provide protection and structure for cells (1). Additionally, specific n-3 FA have critical physiological functions; eicosapentaenoic acid (EPA, 20:5 n-3) and docosahexaenoic acid (DHA, 22:6 n-3) are essential for brain development (1–3). Furthermore, the enrichment of the diets with n-3 FA has been shown to reduce the risk and death from coronary heart disease and cardiovascular diseases (4). Despite the importance of n-3 FA, the body cannot, or is inefficient at biosynthesizing n-3 FA, making diet the only meaningful method to obtain certain beneficial n-3 FA. For instance, alpha-linolenic acid (LNA, 18:3 n-3) is an essential n-3 FA that cannot be biosynthesized by humans and therefore must be obtained by diet. Additionally, DHA and EPA can be biosynthesized from ALA, but this process is inefficient, with 5% of ALA being converted to EPA and <1% into DHA (1–5). As a result of n-3 FA having a key role in human health, and diet being the primary source of certain n-3 FA, a wide variety of functional foods (*i.e*., foods that possess positive human health benefits in addition to basic nutrition) enriched with n-3 FA have become commercially available (5, 6), such as n-3 fortified milk (7, 8) and eggs (9–11).

There are numerous methods to create functional foods fortified with n-3 FA (5, 12). For example, n-3-fortified eggs are produced by supplementing chicken feed with n-3 FA, typically in the form of flax or linseeds (*Linum usitatissimum* L.), which contain a high content of LNA (9–11, 13, 14). As a result of the n-3 FA supplementation, the produced eggs contain up to 5 to 6 times more n-3 FA (primarily LNA) than conventional eggs (11, 15–17). However, methods to create plant-based functional foods fortified with long-chain n-3 FA, are currently limited (5, 18). This limitation leads to the exclusion of a growing group of the population who predominately follow a plant-based diet, such as vegans, vegetarians, pescatarian, and flexiterians from consuming certain functional foods fortified with long-chain n-3 FA (19–21). One method that may be used to develop plant-based n-3 FA fortified functional foods on a commercial scale is nutri-priming, the process of imbibing seeds in a nutrient-rich solution then redrying to their original weight (22–25). Nutri-priming ensures micronutrient availability to the seed and has been shown to improve germination, seedling vigor, resilience, root development, and productivity in multiple crops (22–25). Additionally, in some instances, such as in corn (*Zea mays* L.) (26), chickpea (*Cicer arietinum* L.) (27), and wheat (*Triticum aestivum* L*.)* (27, 28), zinc nutri-priming increased the zinc content of seeds and seedlings (22, 29). Yet, despite the benefits of this fortification approach, there are currently no known examples in the scientific literature of using this process to create n-3 fortified seeds and sprouts.

Therefore, the purpose of this study was to determine whether nutri-priming is a viable option for n-3 fortification for seeds and sprouts. A potential limitation to n-3 FA nutri-priming may be the detrimental effects of n-3 FA on seed germination. Oils, such as crude oil and sunflower oil, have been shown to inhibit the germination and growth of various crops (30–32), likely because the oil forms a hydrophobic film on the seed and its roots, thus, preventing water and gas exchange (32). Consequently, the n-3 FA nutri-priming process may reduce germination and may not be a suitable option for creating n-3 fortified sprouts. The second aim of this study was therefore to determine whether n-3 nutri-priming inhibits germination of primed seeds.

To address these aims, we nutri-primed brown flax in three priming solutions, control (100% deionized water, no fish oil (FO) addition), 10% FO (90% deionized water plus 10% FO), and a 20% FO (80% deionized water plus 20% FO) solution, and determined the FA content and profile in seeds and sprouts as well as their germination percentage. We used flax and FO because they are commonly used to produce n-3 fortified functional foods (33–35) because they contain a high content of total n-3 FA (Σn-3 FA), and, in comparison to FO, a different profile of n-3 FA. Flax is enriched in LNA while FO is enriched with long-chain n-3 FA such as EPA and DHA (5). The utilization of these two sources of n-3 FA should create a functional food with enhanced n-3 FA content and profile that appeals to a wide range of consumers.

## Methods

### Experimental Design

“Brown Flax” (*Linum usitatissimum*) from King's Agriseeds Inc. (Lancaster, PA) was used for the experiment. Eighteen replicates of 1 (±0.05) g of flax seeds (~180 seeds) were primed with either a control, 10%, or a 20% FO-water solution following the procedure described in Holub and Nagpurkar (36). The nutri-priming solution was created by mixing the respective ratio of FO derived from anchovy (Omega-3 Fish Oil EE - 40% EPA and 20% DHA, Jedwards International Inc. Braintree, MA) and deionized water (*e.g*., 1 mL of FO and 4 mL of deionized water for the 20% FO treatment) and vortexing for 10 minutes at 2,500 RPM. Seeds were then primed with the nutri-priming solution using 50 mL conical tubes on an orbital shaker set at 220 RPM for 5 hours. The constant movement by the orbital shaker prevented the separation of the oil and water and ensured that the seeds were in continual contact with the FO. Subsequently, seeds were removed and thoroughly rinsed with deionized water.

After seeds were rinsed, they were left to dry for at least 24 hours at room temperature (22°C). Subsequently, half of the replicates (*n* = 9) were placed in petri dishes (Fisherbrand™ Polystyrene, 100 mm, Pittsburg, PA) lined with three filter papers (Cytiva Whatman™ Qualitative Filter Paper: Grade 1, 90 mm, Maidstone, UK) to undergo sprouting. The remaining 9 replicates were used to quantify the FA content and profile in flax seeds. Filter papers were initially saturated with deionized water and then re-watered *ad libitum* for 10 days. Every 24 hours for 10 days, seeds were scored for germination. A seed was considered germinated if the radicle length reached 3 mm or if cotyledons fully emerged. Once a seed germinated, it was removed from the petri dish and stored at −20°C until further analysis. At the end of 10 days, the total number of germinated seeds and the total number of seeds that failed to germinate were calculated. Additionally, germination percentages were calculated by dividing the number of germinated seeds by the total number of seeds present in the petri dish.

### Fatty Acid Analysis

Fatty acid analysis and calculations of flax seeds and sprouts were conducted as described in Goosen et al. (37) with the exception of using 150 mg of dried sample instead of 500 mg.

### Data Analysis

Absolute FA measures were analyzed using a two-way ANOVA, and multiple comparisons were made using a Tukey HSD test. The two factors used in the analysis were priming solution (control, 10, 20%) and plant stage (seed or sprout). Differences were considered significant with an adjusted *P* < 0.05. Furthermore, a principal component analysis (PCA) was conducted on the absolute FA data (*i.e*., mg/g) using the package “ggfortify” to identify FA that were important in explaining the variability between treatment groups (38). Germination data were analyzed using a general linear model with a beta-regression distribution with the “betareg” function from the R “betareg” package, and multiple comparisons were made using a Tukey HSD test (39). Differences were considered significant with an adjusted *P* < 0.05. All figures were created using the “ggplot2” package in R (40), and all statistical analyses were performed in R version 4.0.2 (41).

## Results

### Fatty Acid Content and Profile

**Figure 1:**
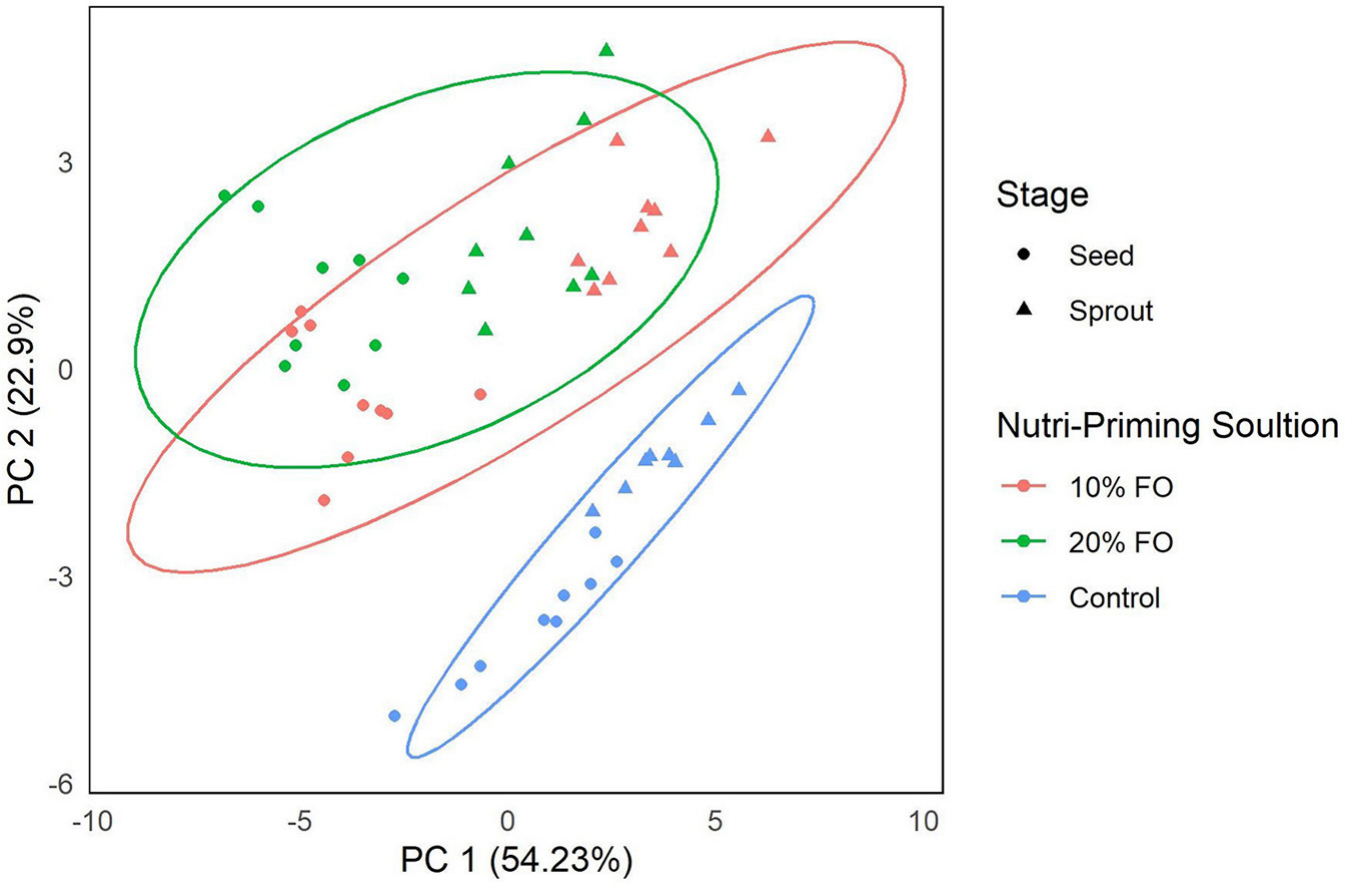
Principal component analysis of FA content (mg per g of sample) of flax seeds and sprouts by nutri-priming solution. Samples are color-coded by nutri-priming treatment (control, 10% fish oil (FO), and 20% FO) and shapes signify plant sage (seed or sprout).

**Figure 2:**
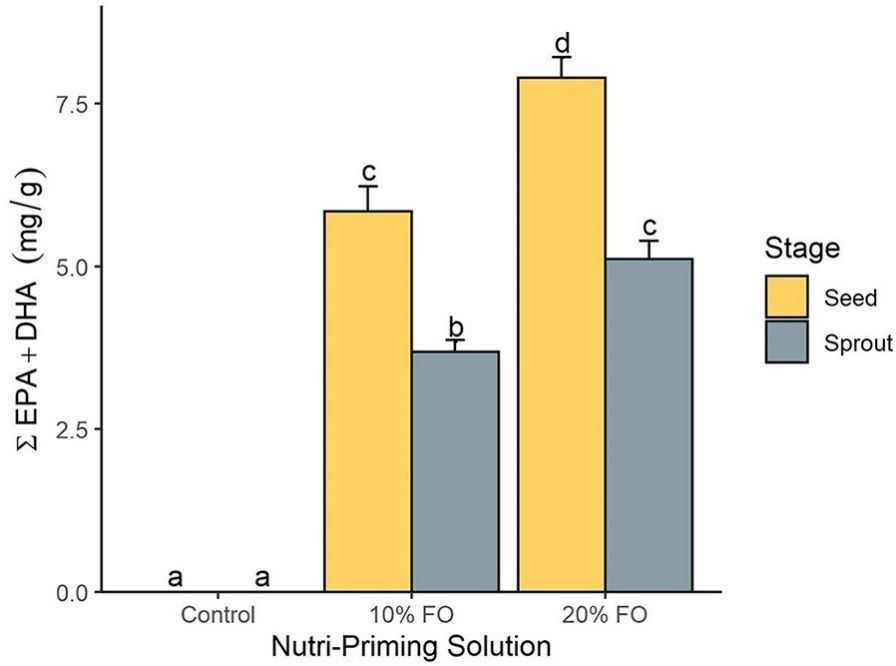
The total content of EPA and DHA (mg per g of sample) by nutri-priming solution and plant stage. Values are expressed as mean and standard error. Means without a common letter differ (*P* < 0.05).

Absolute FA content and profile in seeds and sprouts was influenced by the nutri-priming solution, with an increase of fish-derived FA in the n-3 nutri-primed treatment groups (Table 1). PCA revealed that absolute FA content and profile of nutri-primed seeds and sprouts with 10 and 20% FO addition were similar, with apparent clustering away from the control (Figure 1). The FA driving clustering differences were primarily EPA, DHA, docosapentaenoic acid (DPA, 22:5 n-3), stearidonic acid (SDA, 18:4 n-3), and arachidonic acid (AA, 20:4 n-6) (Supplemental Figure 1). When comparing these FA independently, an increase of absolute content of EPA, DHA, DPA, SDA, and AA was seen in seeds and sprouts for both 10% and 20% FO groups when compared to the control group (Table 1, Figure 2). Furthermore, a dose-response relationship was observed between % FO and EPA, DHA, DPA, and AA (Table 1). As FO percentage increased in the nutri-priming solution, so did the absolute content of EPA, DHA, DPA, and AA; with increases of 34.6, 36.6, 45.5, and 25.8% in seeds, respectively, and 38.8, 37.9, 50, and 33.3% in sprouts, respectively. The content of SDA in seeds remained relatively consistent while the amount of SDA in sprouts increased by 66.7% between treatment groups. Despite increases in fish-derived FA, the absolute content of LA, LNA, total FA (ΣFA), total saturated FA (ΣSFA), total monounsaturated FA (ΣMUFA), ΣPUFA, total n-6 FA (Σn-6 FA), and Σn-3 FA did not increase as a result of n-3 FA nutri-priming (Table 1).

**Table 1:** Key fatty acids (mg/g sample) of flax seeds and sprouts by nutri-priming solution^a^.

	**Nutri-Priming Solution**						******P value******		
**Fatty acid**	**Control**		**10% FO**		**20% FO**		**Nutri-Priming Solution**	**Plant Stage**	**Nutri-Priming Solution X Plant Stage**
	**Seed**	**Sprout**	**Seed**	**Sprout**	**Seed**	**Sprout**			
ΣFA	502.4 ± 16.9	438.9 ± 5.6	524.9 ± 6.2	413.7 ± 7.4	508.9 ± 5.8	432.9 ± 14.9	–	<0.001	–
18:2 *c*9,*c*12 (n-6), LA	78.9 ± 1.8^c^	69.9 ± 1.2^b^	77.7 ± 0.9^c^	64.5 ± 1^a^	76.5 ± 0.6^c^	69.6 ± 1.3^b^	0.022	<0.001	0.026
18:3 *c*9,*c*12,*c*15 (n-3), LNA	289.5 ± 5.8^b^	249.9 ± 4.2^a^	293.1 ± 3.9^b^	227.7 ± 4.4^a^	280.5 ± 3.3^b^	243.9 ± 5.8^a^	–	<0.001	0.008
18:4 *c*6,*c*9,*c*12,*c*15 (n-3), SDA	0 ± 0^a^	0 ± 0^a^	0.16 ± 0.02^d^	0.06 ± 0.01^b^	0.15 ± 0.02^cd^	0.10 ± 0.01^bc^	<0.001	<0.001	0.001
20:4 *c*5,*c*8,*c*11,*c*14 (n-6), AA	0 ± 0^a^	0 ± 0^a^	0.31 ± 0.02^c^	0.21 ± 0.01^b^	0.39 ± 0.02^d^	0.28 ± 0.02^c^	<0.001	<0.001	<0.001
20:5 *c*5,*c*8,*c*11,*c*14,*c*17 (n-3), EPA	0 ± 0^a^	0 ± 0^a^	3.9 ± 0.3^c^	2.5 ± 0.1^b^	5.3 ± 0.2^d^	3.4 ± 0.2^c^	<0.001	<0.001	<0.001
22:5 *c*7,*c*10,*c*13,*c*16,*c*19 (n-3), DPA	0 ± 0^a^	0 ± 0^a^	0.33 ± 0.02^c^	0.18 ± 0.02^b^	0.48 ± 0.02^d^	0.27 ± 0.04^c^	<0.001	<0.001	<0.001
22:6 *c*4,*c*7,*c*10,*c*13,*c*16,*c*19 (n-3), DHA	0 ± 0^a^	0 ± 0^a^	1.9 ± 0.1^c^	1.2 ± 0.06^b^	2.7 ± 0.1^d^	1.7 ± 0.09^c^	<0.001	<0.001	<0.001
ΣSFA	44.0 ± 1^a^	39.1 ± 0.6^b^	45.1 ± 0.5^a^	36.8 ± 0.6^b^	44.1 ± 0.6^a^	39.5 ± 0.8^b^	–	<0.001	0.015
ΣMUFA	99.9 ± 2^a^	84.8 ± 1.2^b^	101.3 ± 0.9^a^	79.7 ± 1.7^b^	100.0 ± 0.7^a^	84.7 ± 2.2^b^	–	<0.001	–
ΣPUFA	369.4 ± 7.5^a^	317.7 ± 5.2^b^	378.5 ± 4.9^a^	297.2 ± 5.4^b^	367.1 ± 3.9^a^	320.5 ± 6.8^b^	–	<0.001	0.008
Σn-6 FA	79.4 ± 1.8^c^	70.5 ± 1.2^b^	78.6 ± 0.9^c^	65.2 ± 1^a^	77.5 ± 0.6^c^	70.5 ± 1.3^b^	0.038	<0.001	0.026
Σn-3 FA	289.9 ± 5.8^a^	247.3 ± 4.2^b^	299.9 ± 3.9^a^	232.1 ± 4.4^b^	289.6 ± 3.3^a^	249.9 ± 5.6^b^	–	<0.001	0.007

a * Values are expressed as mean ± standard error of the mean; values within a row without a common letter differ by nutri-priming solution and plant stage (Nutri-priming Solution X Plant Stage)*.

Plant stage (*i.e*., seed and sprout) influenced the absolute FA content, with sprouts having a consistently lower FA content than their seed counterparts in all treatment groups (Table 1, Figure 2). For instance, in the 20% FO treatment group, EPA, DHA, DPA, AA, SDA, ALA, and LA decreased by 35.3, 35.5, 43.8, 28.2, 33.3, 13, and 9.1%, respectively. Similar losses were observed in the control and 10% FO group (Table 1).

### Flax Germination

**Figure 3:**
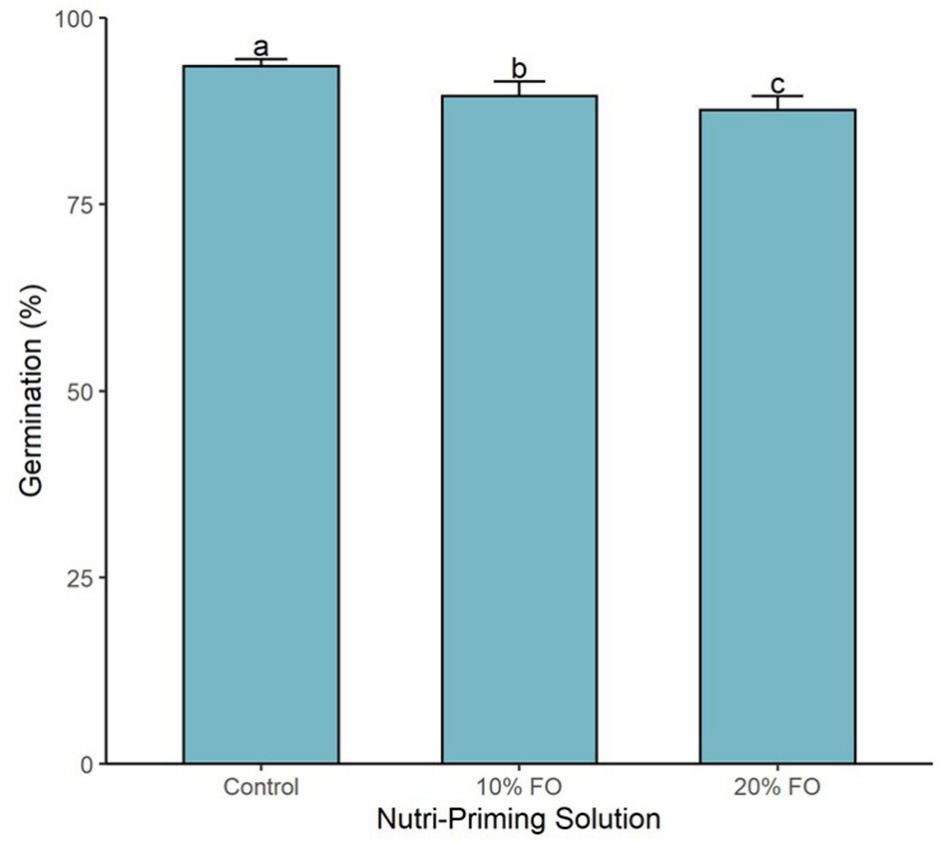
Germination percentage of sprouts by nutri-priming solution. Values are expressed as mean and standard error. Means without a common letter differ (*P* < 0.05).

Germination percentage was affected by the nutri-priming solution (*P* < 0.001, Figure 3). As the inclusion of FO% increased in the nutri-priming solution, germination percentage decreased (Figure 3). The highest inclusion rate (*i.e*., 20% FO) resulted in the lowest germination percentage with 87.7% (±0.6), followed by FO 10% with 89.5% (±0.7), and then the control group with 93.5% (±0.3) (Figure 3).

## Discussion

We sought to determine whether n-3 nutri-priming is a viable option for the fortification of flax seeds and sprouts. Additionally, we assessed whether n-3 nutri-priming inhibits germination of nutri-primed seeds. We found that n-3 nutri-priming with FO influenced FA content and profile in seeds and sprouts (Table 1). Specifically, we observed an increase in the absolute content of EPA, DHA, DPA, SDA, and AA due to FO nutri-priming (Figure 2, Table 1). Additionally, as the percent of FO increased in the nutri-priming solution, so did the absolute content of EPA, DHA, DPA, and AA in both the seeds and sprouts (Figure 2, Table 1). This increase was expected, as flax does not contain EPA, SDA, DHA, DPA, and AA, while FO contains a moderate to high amount of each of these FA (5, 33, 34, 42, 43). In contrast, n-3 FA nutri-priming did not increase the absolute content of LA, LNA, ΣFA, ΣSFA, ΣMUFA, ΣPUFA, and Σn-6 FA and Σn-3 FA (Table 1). The primary reason for this is that flax is naturally high in Σn-3 FA, primarily LNA. We found that flax comprised of approximately 290 mg LNA per gram sample, which accounts for 56% of the total FA content (Supplemental Table 1). This finding is consistent with other studies that demonstrated flax to contain 39.9% to 60.4% of LNA (5, 33, 34). Because of flax having a high amount of LNA, the moderate increase in other n-3 FA, such as EPA, DHA, DPA, and SDA, did not significantly increase the overall content of total n-3 FA in seeds or sprouts. Potentially, n-3 FA nutri-priming with a higher percentage of FO or using other seeds with a low to moderate total n-3 FA content, such as mung bean (44), sunflower (45, 46), sesame (46), or lentil (45), may result in an increased content of ΣPUFA and Σn-3 FA.

The second objective of our study was to assess whether n-3 FA nutri-priming inhibits germination. We found that n-3 FA nutri-priming decreased the germination percentage of primed flax seeds. The decrease in germination percentage, while significant, is modest when compared to studies that assessed the effect of the presence of soil oils on crop germination (30–32). For instance, sunflower oil decreased wheat germination by 20% (32), and crude oil decreased corn germination by 37.5 to 93.8% (30). Further research utilizing different seed types is required to determine whether the reduction in germination percentage is a flax-specific or a general trend associated with n-3 FA nutri-priming.

Our results also indicate that n-3 nutri-primed seeds and sprouts can be used as a functional food to increase EPA and DHA in diets. The American Heart Association recommends consuming two fatty fish servings per week, which amounts to approximately 250 mg of EPA and DHA per day (47). Similarly, the World Health Organization (48), Food and Agriculture Organization (48), the Dietary Guidelines for Americans (49), and the European Food Safety Authority (50) recommend that adults consume 250 mg of EPA and DHA per day. To reach the international dietary recommended amount of EPA and DHA, one would need to consume 31.6, 48.9, 42.8, or 67.6 g of the 20% FO-treated seeds, 20% FO-treated sprouts, 10% FO-treated seeds, and 10% FO-treated sprouts, respectively. For the 20% FO-treated seed treatment group, which contained the highest combined EPA and DHA content (Table 1), the consumption of 31.6 grams of flax seed exceeds the Flax Council of Canada recommendation of consuming 8–16 g of flax per day and the daily recommended amount (1.1–1.6 grams per day) of LNA by Dietary Guidelines for Americans (49, 51). However, the consumption of more than 16 g of flax is safe and may be beneficial for human health (52). Cunnane et al. (53) concluded that consuming 50 g of flax per day was palatable, safe, and beneficial to human health by increasing n-3 FA in blood plasma and erythrocytes and reducing postprandial glucose response. Additionally, the consumption of 30 g of milled flaxseed every day for 6 months decreased systolic and diastolic blood pressure in patients with peripheral arterial disease (54), while the daily consumption of 40 g of milled flax seeds reduced cholesterol levels (51). Lastly, no clinical trial has reported toxicity due to dietary supplementation of flax (52). Therefore, the daily consumption of 30 to 50 g of n-3 nutri-primed flax seeds and sprouts is most likely a feasible and safe amount of flax to consume.

Other potential drawbacks including areas of future studies for n-3 nutri-priming are the sustainability and acceptability of FO, the cost-effectiveness of this approach, the commercial application of this process, and the effects of n-3 nutri-priming on the sensory and nutritional components of flax seeds and sprouts. The sustainability of FO has been called into question due to the rapid decline of fish stocks from overfishing and climate change (55–57). A typical FO supplement contains 1,000 mg of FO, which translates to 300 mg of EPA and DHA (58, 59). n-3 nutri-priming with 20% FO solution would utilize at least 25 times more FO to deliver the same amount of EPA and DHA as a typical FO supplement. Therefore, the results of this study indicate that n-3 nutri-priming with FO may be less cost-effective at increasing EPA and DHA in diets than typical FO supplements. Another major problem is that FO is animal-based which makes it unappealing to some consumers, like vegetarians and vegans, and even to some omnivores due to its fishy taste and odor (12, 33, 43, 60). Therefore, the effect of n-3 nutri-priming on the sensory components of flax seeds and sprouts needs to be thoroughly evaluated through sensory evaluation studies to gauge consumer acceptability. To alleviate these potential drawbacks, n-3 nutri-priming with alternative plant-based sustainable oils, such as echium oil (*Echium plantagineum*), may be a possible solution (61). Echium oil is a neutral, plant-based, and sustainable source of n-3 FA, primarily SDA (13–14% of total FA) (61, 62). While echium oil does not contain EPA or DHA, it has a high amount of SDA (an intermediate in the biosynthetic conversion of LNA to EPA), which the body can readily convert to EPA (43, 62). Most importantly, echium oil and specifically SDA show similar health benefits as FO, EPA, and DHA (43, 63–66). Furthermore, additional studies are required to understand the long-term functional stability of n-3 FA fortified seeds and sprouts and to determine if there are any effects on other nutritional components such as protein, carbohydrate, fiber, and antinutrient content. The long-term stability of the n-3 FA primed seeds and sprouts could be a major concern as n-3 PUFA are highly prone to oxidative degradation (67). Incorporating an antioxidant, such as vitamin E, into the n-3 FA nutri-priming process may mitigate this concern and extend the shelf life of n-3 FA fortified seeds and sprouts.

## Conclusion

We evaluated the efficacy of n-3 nutri-priming of flax seeds and sprouts to increase n-3 FA and determined if this process inhibited germination. We demonstrated that FO nutri-priming of flax modified the FA profile of flax seeds and sprouts with the inclusion of beneficial FA, specifically EPA, DHA, DPA, SDA, and AA. Nutri-priming, however, did not increase the total content of n-3 FA of flax. This was because the modest increase in FO-derived n-3 FA, such as EPA, DHA, DPA, and SDA did not offset the naturally large amount of LNA present in flax. Additionally, our results also demonstrate that nutri-priming decreases germination. Therefore, n-3 nutri-priming does not seem to be a viable option for n-3 fortification of flax seeds or sprouts. However, further research utilizing other seed types, oil types, and oil concentrations is required to fully determine whether nutri-priming is indeed a viable commercial method for creating plant-based n-3 fortified functional foods.

## Data Availability Statement

The raw data supporting the conclusions of this article will be made available by the authors, without undue reservation.

## Author Contributions

EM conducted the study and wrote the manuscript. JK and HD designed the study, edited, and reviewed the manuscript. All authors contributed to the article and approved the submitted version.

## Conflict of Interest

This research was partially funded through Botanical Intelligence LLC, the current patent holder of Method of fortifying seeds with an essential fatty acid, fortified seed and food product (WO 2005/065468 A1). The funder was not involved in the study design, collection, analysis, interpretation of data, the writing of this article or the decision to submit it for publication. The authors declare that the research was conducted in the absence of any commercial or financial relationships that could be construed as a potential conflict of interest.

## Publisher's Note

All claims expressed in this article are solely those of the authors and do not necessarily represent those of their affiliated organizations, or those of the publisher, the editors and the reviewers. Any product that may be evaluated in this article, or claim that may be made by its manufacturer, is not guaranteed or endorsed by the publisher.

## Abbreviations

FO: fish oil
LA: linoleic acid
LNA: alpha-linolenic acid
DHA: docosahexaenoic acid
EPA: eicosapentaenoic acid
DPA: docosapentaenoic acid
SDA: stearidonic acid
AA: arachidonic acid
FA: fatty acid
MUFA: monounsaturated fatty acids
n-3 FA: n-3 fatty acids
n-6 FA: n-6 fatty acids
PUFA: polyunsaturated fatty acids
SFA: saturated fatty acids.
