# Supplementary material for: Omega-3 Fatty Acid Fortification of Flax Through Nutri-Priming
Source: Front Nutr. 2021 Aug 20;8:715287. doi: 10.3389/fnut.2021.715287 (PMC8417600; doi:10.3389/fnut.2021.715287)
Supplement: Supplementary file 1 [file Data_Sheet_1.PDF]

Supplementary Material

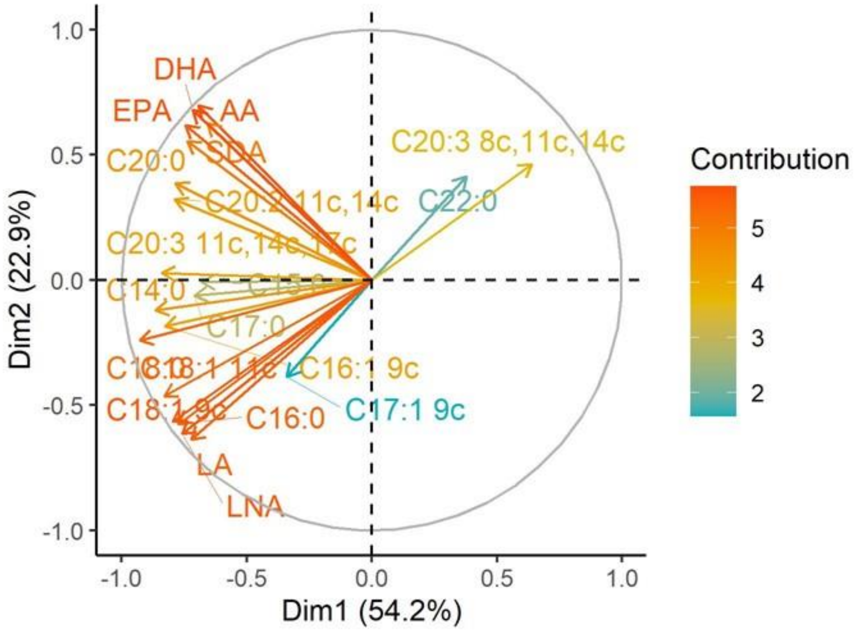

Supplemental Figure S1. Principal component analysis of FA content (mg per g of sample) of flax seeds and sprouts by nutri-priming solution showing the loadings of each variable. Color and length of arrows signify the contribution of that variable (fatty acid) in explaining the variability in the data set.

Supplemental Table S1. FA (g/100 g of total fatty acids) of flax seeds and sprouts by nutri-priming solution[a]

	Nutri-Priming Solution					
**Fatty acid**	**Control**		**10% FO**		**20% FO**	
	**Seed**	**Sprout**	**Seed**	**Sprout**	**Seed**	**Sprout**
14:0	0.08 ± 0.01	0.08 ± 0.00	0.08 ± 0.00	0.08 ± 0.01	0.08 ± 0.00	0.08 ± 0.01
15:0	0.03 ± 0.00	0.03 ± 0.00	0.03 ± 0.00	0.03 ± 0.01	0.04 ± 0.00	0.03 ± 0.01
16:0	4.93 ± 0.05	4.88 ± 0.06	4.86 ± 0.06	4.86 ± 0.08	4.86 ± 0.04	4.88 ± 0.07
16:1 c9	0.09 ± 0.00	0.09 ± 0.00	0.09 ± 0.01	0.09 ± 0.01	0.1 ± 0.01	0.1 ± 0.01
17:0	0.05 ± 0.01	0.06 ± 0.01	0.06 ± 0.00	0.07 ± 0.01	0.07 ± 0.01	0.06 ± 0.00
17:1 *c*9	0.02 ± 0.00	0.02 ± 0.01	0.02 ± 0.00	0.02 ± 0.00	0.02 ± 0.01	0.02 ± 0.00
18:0	3.23 ± 0.09	3.47 ± 0.06	3.28 ± 0.04	3.48 ± 0.08	3.36 ± 0.04	3.45 ± 0.06
18:1 *c*9	18.60 ± 0.51	18.37 ± 0.39	18.37 ± 0.30	18.37 ± 0.55	18.66 ± 0.24	18.38 ± 0.51
18:1 *c*11	0.73 ± 0.02	0.74 ± 0.02	0.79 ± 0.02	0.78 ± 0.02	0.78 ± 0.02	0.78 ± 0.02
18:2 *c*9,*c*12 (n-6), LA	15.50 ± 0.41	15.83 ± 0.36	14.81 ± 0.11	15.54 ± 0.21	14.96 ± 0.23	15.64 ± 0.29
18:3 *c*9,*c*12,*c*15 (n-3), LNA	56.31 ± 0.65	55.91 ± 0.73	55.82 ± 0.30	55.04 ± 0.58	54.82 ± 0.5	54.79 ± 0.68
18:4 *c*6,*c*9,*c*12,*c*15 (n-3), SDA	0 ± 0	0 ± 0	0.03 ± 0.01	0.01 ± 0.00	0.03 ± 0.01	0.02 ± 0.01
20:0	0.13 ± 0.01	0.15 ± 0.01	0.15 ± 0.01	0.17 ± 0.01	0.16 ± 0.01	0.17 ± 0.01
20:2 11*c*,14*c* (n-6)	0.03 ± 0.00	0.03 ± 0.00	0.04 ± 0.01	0.03 ± 0.01	0.04 ± 0.01	0.04 ± 0.01
20:3 8*c*,11*c*,14*c* (n-6)	0.07 ± 0.00	0.11 ± 0.01	0.07 ± 0.00	0.13 ± 0.02	0.07 ± 0.01	0.12 ± 0.02
20:3 11*c*,14*c*,17*c* (n-3)	0.08 ± 0.01	0.08 ± 0.01	0.09 ± 0.01	0.09 ± 0.01	0.09 ± 0.01	0.09 ± 0.01
20:4 *c*5,*c*8,*c*11,*c*14 (n-6), AA	0 ± 0	0 ± 0	0.06 ± 0.01	0.05 ± 0.01	0.08 ± 0.01	0.06 ± 0.01
20:5 *c*5,*c*8,*c*11,*c*14,*c*17 (n-3), EPA	0 ± 0	0 ± 0	0.65 ± 0.03	0.57 ± 0.06	1.03 ± 0.11	0.77 ± 0.18
21:5 *c*6, *c*9, *c*12,*c*15, *c*18 (n-3)	0 ± 0	0 ± 0	0.01 ± 0.00	0.01 ± 0.00	0.02 ± 0.01	0.02 ± 0.01
22:0	0.14 ± 0.01	0.18 ± 0.01	0.14 ± 0.00	0.20 ± 0.01	0.14 ± 0.01	0.19 ± 0.01
22:5 *c*7,*c*10,*c*13,*c*16,*c*19 (n-3), DPA	0 ± 0	0 ± 0	0.06 ± 0.01	0.04 ± 0.02	0.09 ± 0.01	0.06 ± 0.03

22:6 *c*4,*c*7,*c*10,*c*13,*c*16,*c*19 (n-3), DHA	0 ± 0	0 ± 0	0.37 ± 0.07	0.3 ± 0.05	0.52 ± 0.07	0.39 ± 0.08
**Fatty acid Classes**						
Σ SFA	8.59 ± 0.08	8.84 ± 0.09	8.60 ± 0.06	8.89 ± 0.12	8.71 ± 0.07	8.88 ± 0.09
Σ MUFA	19.43 ± 0.52	19.21 ± 0.41	19.29 ± 0.31	19.26 ± 0.56	19.55 ± 0.25	19.28 ± 0.51
Σ PUFA	71.99 ± 0.54	71.95 ± 0.46	72.00 ± 0.26	71.82 ± 0.57	71.74 ± 0.31	71.99 ± 0.62
Σ n-6 FA	15.6 ± 0.41	15.96 ± 0.36	14.97 ± 0.11	15.76 ± 0.22	15.14 ± 0.23	15.86 ± 0.31
Σ n-3 FA	56.39 ± 0.65	55.99 ± 0.73	57.03 ± 0.28	56.06 ± 0.51	56.6 ± 0.43	56.14 ± 0.59

[a]Values are expressed as mean ± standard deviation of the mean.
